# FlhF Is Required for Swarming Motility and Full Pathogenicity of *Bacillus cereus*

**DOI:** 10.3389/fmicb.2016.01644

**Published:** 2016-10-19

**Authors:** Diletta Mazzantini, Francesco Celandroni, Sara Salvetti, Sokhna A. Gueye, Antonella Lupetti, Sonia Senesi, Emilia Ghelardi

**Affiliations:** ^1^Department of Translational Research and New Technologies in Medicine and Surgery, University of PisaPisa, Italy; ^2^Department of Biology, University of PisaPisa, Italy; ^3^Research Center Nutraceuticals and Food for Health-Nutrafood, University of PisaPisa, Italy

**Keywords:** FlhF, *Bacillus cereus*, swarming, protein secretion, virulence

## Abstract

Besides sporulation, *Bacillus cereus* can undergo a differentiation process in which short swimmer cells become elongated and hyperflagellated swarmer cells that favor migration of the bacterial community on a surface. The functionally enigmatic flagellar protein FlhF, which is the third paralog of the signal recognition particle (SRP) GTPases Ffh and FtsY, is required for swarming in many bacteria. Previous data showed that FlhF is involved in the control of the number and positioning of flagella in *B. cereus*. In this study, *in silico* analysis of *B. cereus* FlhF revealed that this protein presents conserved domains that are typical of SRPs in many organisms and a peculiar N-terminal basic domain. By proteomic analysis, a significant effect of FlhF depletion on the amount of secreted proteins was found with some proteins increased (e.g., B component of the non-hemolytic enterotoxin, cereolysin O, enolase) and others reduced (e.g., flagellin, L_2_ component of hemolysin BL, bacillolysin, sphingomyelinase, PC-PLC, PI-PLC, cytotoxin K) in the extracellular proteome of a Δ*flhF* mutant. Deprivation of FlhF also resulted in significant attenuation in the pathogenicity of this strain in an experimental model of infection in *Galleria mellonella* larvae. Our work highlights the multifunctional role of FlhF in *B. cereus*, being this protein involved in bacterial flagellation, swarming, protein secretion, and pathogenicity.

## Introduction

*Bacillus cereus* is a Gram-positive, motile, spore-bearing rod, frequently isolated from the soil, where the spore ensures its persistence under adverse conditions. Long known as agent of food-borne diseases, this organism is now recognized to be able to cause local and systemic infections in humans (Bottone, 2010; Logan, 2012; Celandroni et al., 2016). The pathogenic potential of this bacterium is related to the secretion of several virulence proteins, e.g., hemolysins, phospholipases, trimeric toxins (hemolysin BL, HBL; non-hemolytic enterotoxin, NHE), cytotoxin K (CytK), proteases (Senesi and Ghelardi, 2010; Ramarao and Sanchis, 2013; Jeßberger et al., 2015), and to motility modes, such as swimming and swarming (Senesi et al., 2010; Celandroni et al., 2016). Bacterial swarming is a flagellum-driven social form of locomotion in which cells undergo a periodical differentiation process leading to the production of long and hyperflagellated elements, the swarmer cells, which coordinately migrate across surfaces (Kearns, 2010; Partridge and Harshey, 2013). Swarming confers an advantage for the colonization of natural and host surfaces and can contribute to bacterial virulence. Notably, swarming increases HBL secretion by *B. cereus* (Ghelardi et al., 2007) and enhances the pathogenicity of this bacterium in an experimental endophthalmitis model (Callegan et al., 2006).

In a previous study, we demonstrated that the protein FlhF plays a major role in controlling the arrangement of flagella in *B. cereus* (Salvetti et al., 2007). The proteins FlhF and FlhG are essential for establishing correct place and quantity of flagella in many but not all bacterial species (Schniederberend et al., 2013). In *Bacillus subtilis*, FlhF and FlhG behave as two antagonistic proteins regulating the pattern of flagellar basal body formation to control flagella localization, but they do not influence the number of flagella on the cell surface (Guttenplan et al., 2013). In *B. cereus*, no FlhG homologue was found and the lack of FlhF, other than leading to mislocalization of flagella, causes a reduction in the number of flagella and reduces swimming migration (Salvetti et al., 2007).

FlhF belongs to the GTP-binding signal recognition particle (SRP) family, which also includes Ffh and FtsY (Bange et al., 2007; Schuhmacher et al., 2015). SRP-GTPases are required for the cotranslational targeting of many proteins to the membrane through the recognition and binding of their N-terminus signal peptide during protein synthesis. Nevertheless, FlhF appeared not to be involved in protein secretion mechanisms in *B. subtilis* (Zanen et al., 2004). Differently, in *Pseudomonas putida* and *B. cereus*, the FlhF depletion altered the profile of secreted proteins (Pandza et al., 2000; Salvetti et al., 2007). In particular, a Δ*flhF* mutant of *B. cereus* showed an increase in the extracellular levels of NHE and a decrease in HBL and phosphatidyl-choline specific phospholipase C (PC-PLC) (Salvetti et al., 2007). Thus, the aim of the present study was to gain more insight into the function of FlhF in *B. cereus* by evaluating the effects of FlhF depletion on interconnected cellular functions such as swarming, protein secretion, and virulence, which may all depend from protein targeting to the membrane.

## Materials and Methods

### Bacterial Strains and Growth Conditions

*Bacillus cereus* ATCC 14579 wild type (wt), its *flhF* (GeneBank ID: *Bc1670*) mutant derivative (Δ*flhF*, MP06), and complemented strain (Δ*flhF*-comp, MP07) (Salvetti et al., 2007) stocks from -80° C were streaked on brain heart infusion supplemented with 0.1% (w/v) glucose (BHIG; Becton Dickinson, Cockeysville, NJ, USA) plates and incubated at 37° C. BHIG was also used for liquid cultures. When required, 5 μg/ml erythromycin for strain MP06 or 30 μg/ml kanamycin for strain MP07 were added to the media. MP07 cultures were also supplemented with 4 mM isopropil-β-D-1-tiogalattopiranoside (IPTG; Sigma–Aldrich, St. Louis, MO, USA) in order to induce *pspac* dependent gene expression.

### *In silico* Analysis

BLAST^1^ was used for comparative analysis of nucleotide and protein sequences. Protein sequences in the FASTA format were retrieved from the UniProt database^2^ (The UniProt Consortium, 2015). Functional domain analysis was performed using the ProDom Server^3^ (Bru et al., 2005). The presumptive secondary and tridimensional structure of proteins were generated using the Phyre2 web portal for protein modeling, prediction and analysis^4^ (Kelley et al., 2015) and the Raptor X Structure Prediction Server^5^ (Källberg et al., 2012), respectively.

### Swarming Motility

For each experiment, swarm plates (TrA plates; 1% tryptone, 0.5% NaCl, 0.7% granulated agar) were prepared fresh daily and allowed to sit at room temperature overnight before use (Salvetti et al., 2011). Swarming was initiated by spotting 50 μl of a culture containing approximately 2×10^4^ cells/ml onto the center of TrA plates, and incubating cultures at 37° C. Swarming migration was evaluated by measuring colony diameters after 8 h. Since *B. cereus* flagella are very fragile, bacterial samples were taken by slide overlay of single agar blocks (5 mm ×5 mm) that contained different colony portions. Bacterial cells were stained with tannic acid and silver nitrate (Harshey and Matsuyama, 1994) for microscopy. Several samples were analyzed at 1000× magnification using an optical microscope (BH-2; Olympus, Tokyo, Japan). All experiments were performed in duplicate in three separate days.

### Preparation of Culture Supernatants

Protein samples were prepared by growing bacterial cells to the late exponential growth phase in BHIG at 200 rpm for 6 h at 37° C. Culture supernatants were collected by high-speed centrifugation (10000 × *g*), added with the serine protease inhibitor phenylmethylsulfonyl fluoride 1 mM (PMSF; Sigma–Aldrich), and stored at -80° C until use. Protein concentration in supernatants was determined by the bicinchoninic acid (BCA) assay (Smith et al., 1985), using bovine serum albumin as a standard. The activity of fructose-1,6-bisphosphate aldolase in culture supernatants was spectrophotometrically measured at 30° C by following the rate of NADH oxidation at 340 nm according to the method described by Warth (1980).

### Two-Dimensional Electrophoresis (2-DE)

Proteins contained in supernatants were precipitated using the TCA method (trichloroacetic acid, Sigma–Aldrich). Precipitated proteins were washed eight-times with cold 96% (v/v) ethanol, air-dried and suspended in sample rehydration buffer (7 M urea, 2 M thiourea, 4% CHAPS, 2.4% aminosulfobetaine-14; Invitrogen, Carlsbad, CA, USA). ZOOM^®^ IPG strips with a linear pH range of 4–7 (Invitrogen) were rehydrated for 16 h in 400 μl of sample rehydration buffer and 10 μg of protein samples were subsequently added by cup loading. Focusing was carried out at 200 V for 20 min, 450 V for 15 min, 750 V for 15 min, and 2000 V for 30 min using the Xcell SureLock^TM^ Mini-Cell system (Invitrogen). The IPG strips were then equilibrated for 10 min in 5 ml of equilibration solution (LDS 1X; DTT 0.5 mM). For the second dimension, samples were run at 200 V for 35 min on 4–12% gradient SDS-PAGE gel (Bis-Tris ZOOM^TM^ Gel, 1.0 mm IPG-well; Invitrogen). Three independent biological experiments were performed in separate days. Gels were silver or Coomassie Blue (Sigma–Aldrich) stained, according to standard procedures. Gels were scanned at 300 dpi and 8 bits depth on an Image Scanner equipped with a film-scanning unit and analyzed with the Image-Master 2D Platinum v.6 software (GE Healthcare, Little Chalfont, UK). The spots were quantified after normalization and spot volumes (pixel intensity × area) were expressed as percentage of the total volume of the spots on the gel. Presumptive analysis of protein gels was performed by comparison of silver stained 2-DE gels with gels available in the literature (Gohar et al., 2002, 2005) and then protein spots were identified by spot excision from Coomassie-Blue stained gels and subsequent identification. To this purpose, excided spots were digested with 0.1–0.5 μg of trypsin at 37° C for 6 h (Shevchenko et al., 1996). Digested proteins were analyzed by liquid chromatography coupled with tandem mass spectrometry (LC-MS/MS) at Université de Genève, Proteomics Core Facility (Genève, CH). Database searches were performed with the Mascot Server (Matrix Science Ltd; version 04_2014^6^) and results were analyzed and validated using the Scaffold software (Proteome Software Inc.). Searches were performed using trypsin for the fragments cleavage, allowing up to one trypsin miscleavage and without restriction on protein mass or isoelectric point (p*I*). Fixed modifications included carbamidomethylation of cysteine, while variable modifications comprised oxidation of methionine. Peptide masses ranged from 849.0 m/z to 4001.0 m/z with a charge (z) of 1 +. The mass tolerance was set to ± 25 ppm. Significance was established according to expectancy (e) value transformed into MS score (with significance at a *P*-value <0.05 at scores over 70), percentage coverage and theoretical p*I* and molecular weight (Mw) compared to the approximate experimental values observed on 2-DE gels.

Identified proteins were classified based on their biological functions using the Kyoto Encyclopedia of Genes and Genomes (KEGG) database resource^7^. Protein sequences were analyzed using the SIGNALIP 4.1 Server^8^, TATP 1.0^9^, SecretomeP 2.0 Server^10^ and TMpred program^11^ in order to evaluate the presence of Sec-type signals, Tat-type signals, non-classically secretion signals or transmembrane domains, respectively.

### Insects and Infection Experiments

*Galleria mellonella* larvae, obtained from Mous Live Bait (Balk, Netherland), were selected by weight (0.2–0.3 g) and absence of dark spots on the cuticle. Bacteria were grown to the late exponential growth phase in BHIG at 37° C for 6 h and harvested by centrifugation at 5000 × *g* for 10 min. Bacterial suspensions were prepared in phosphate buffered saline (PBS: 1 M KH_2_PO_4_, 1 M K_2_HPO_4_, 5 M NaCl, pH 7.2) and bacteria counted using a hemocytometer. Three infectious doses (about 10^3^, 10^2^, and 10^1^ CFU per larva) were used to infect three groups of 20 larvae. Larva abdomen was accurately cleaned with 70% ethanol and 10 μl of bacterial suspension was injected into the hemocoel through the last right pro-leg, with a sterile Hamilton syringe (Sigma–Aldrich) via a 26-gage needle. A control group of larvae was injected with PBS only. Infectious doses were checked by CFU count after plating appropriate dilutions and incubating 16 h at 37°C. Infected larvae were kept at 37°C and mortality was recorded after 24 h. Each experiment was performed three times in separate days.

To assess bacterial ability to multiply *in vivo*, groups of 20 animals were infected with 10^4^ CFU/larva of *B. cereus* wt, Δ*flhF*, or Δ*flhF*-comp. Groups of three insects were collected at different times post infection (2, 4, 6, 8, and 24 h) and their surfaces were disinfected with 70% ethanol. Larvae were homogenized in 2 ml of PBS using a Stomacher 400 Circulator (Seward, Worthing, UK). Serial dilutions of the homogenates were plated on LB agar and colonies were counted after incubation at 37°C for 24 h. Non-infected larvae were used as negative control. Three independent biological replicates were performed.

### Statistical Analysis

Data were expressed as the mean ± SD. A *P*-value <0.05 was considered significant. For 2-DE experiments, the One-way Anova analysis and the two-tailored Student’s *t*-test for equal or unequal variance were applied. For *G. mellonella* experiments, the 50% lethal dose (LD_50_) values were estimated by probit analysis (Finney, 1971). Differences in mortality rates obtained for each infectious dose and in the CFU number for each time tested were evaluated by the One-way Anova analysis.

## Results

### *In silico* Analysis of *B. cereus* FlhF

By BLAST alignments, the *flhF* nucleotide sequence resulted to be highly conserved (from 90 to 100%; Score ≥200) among different *B. cereus* strains. In a previous work (Salvetti et al., 2007), we showed that *B. cereus* FlhF possesses a C-terminal G domain, that is strongly conserved among SRP-GTPases (Zanen et al., 2004; Bange et al., 2007; Salvetti et al., 2007; Balaban et al., 2009; Green et al., 2009; Schniederberend et al., 2013; Schuhmacher et al., 2015), and a less conserved N-terminal B domain. Herein, revisited analysis with the ProDom program reveals a nucleoside-triphosphatase (amino acids 176–219), an SRP receptor (amino acids 220–302), an SRP54-type protein GTPase (amino acids 303–353), and an SRP-dependent cotranslational protein-membrane targeting (amino acids 354–439) subdomain inside the NG domain of *B. cereus* FlhF. Unlike the FlhF proteins of *Vibrio cholerae. P. aeruginosa*, and *B. subtilis* in which the N-terminus comprises a GTPase activity subdomain, the N-terminus (B domain) of *B. cereus* FlhF is unique and functionally unknown.

**FIGURE 1:**
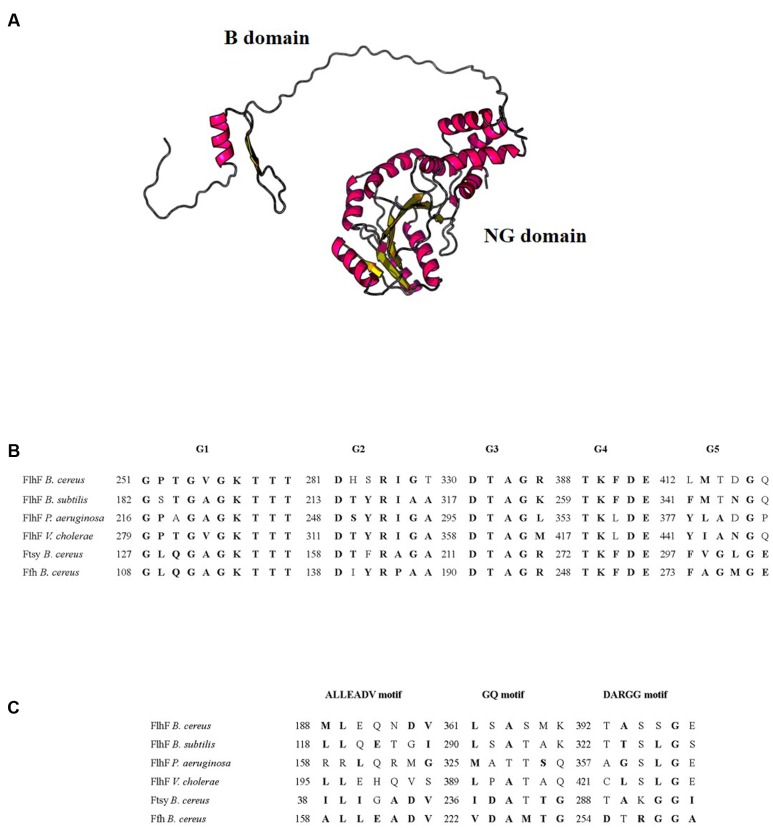
***In silico* analysis of *B. cereus* FlhF.** **(A)** Model of the three-dimensional structure of *B. cereus* FlhF. **(B)** Alignment of the amino acid sequences of the G1–G5 signatures of the G domain and their respective positions. **(C)** Alignment of the amino acid sequences of the ALLEADV, GQ and DARGG motifs and their respective positions. Conserved amino acids belonging to the same chemical class are marked in bold.

We used the Phyre2 program (Kelley et al., 2015) and the Raptor X Structure Prediction Server (Källberg et al., 2012) in order to define the putative secondary and three-dimensional structure of *B. cereus* FlhF. The protein appeared to be constituted by 45% α-helices and 30% β-sheets and to consist of two distinct portions connected by an unstructured peptide linker (**Figure 1A**). The B domain was predicted by the Raptor X Structure Prediction Server only, with a *P*-value of 2.5 × 10^-2^, while the NG domain was predicted by both programs with a good *P*-value (7.43 × 10^-7^ for Raptor X Structure Prediction Server and 100.0% confidence by the single highest scoring template with Phyre2) and it is 100% similar in structure to *B. subtilis* FlhF.

In all described SRP-GTPases, five signature elements (G1–G5) interacting with the GDP/GTP-Mg^2+^ complex have been identified in the G domain (Anand et al., 2006; Bange et al., 2007). These elements have never been described in *B. cereus* SRPs. As shown in **Figure 1B**, the alignment between the FlhF G1–G5 signatures of *B. subtilis, P. aeruginosa*, and *V. cholerae* with the amino acid sequence of *B. cereus* FlhF, FtsY, and Ffh revealed that all these signatures are also present inside the G domain of *B. cereus* SRP-GTPases. In particular, G3 and G4 signatures are identical among FlhF, Ffh, and FtsY of *B. cereus* and G1 is highly conserved in all the analyzed bacterial species. The G2 signature of *B. cereus* SRP-GTPases are less conserved compared to the same sequence of *B. subtilis. P. aeruginosa*, and *V. cholerae*, while G5 is generally less conserved among all the species analyzed.

Ffh and FtsY of many organisms contain additional sequence motifs (ALLEADV in the N domain; GQ and DARGG in the G domain) that are essential for the communication between functional domains (Bange et al., 2007). Since these motifs have never been described in *B. cereus* SRPs, we first identified them in Ffh and FtsY of *B. cereus*, through amino acid alignments with the *Escherichia coli* and *B. subtilis* orthologs (data not shown). **Figure 1C** shows the alignment between the ALLEADV, GQ and DARGG motifs in *B. cereus* SRPs and the putative corresponding regions of FlhF in *B. subtilis*, *P. aeruginosa*, and *V. cholerae*.

### FlhF and Swarming Motility

**FIGURE 2:**
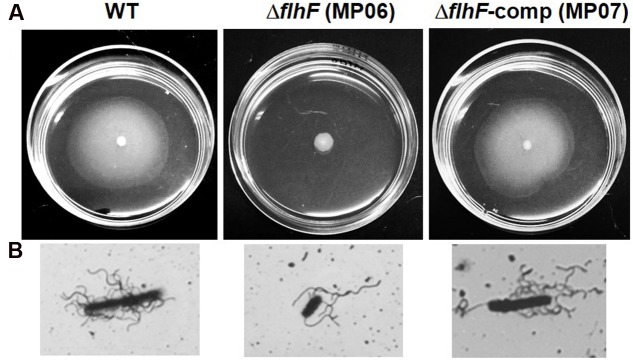
**Analysis of swarming motility.** **(A)** Colonies produced on swarming TrA plates. **(B)** Representative cells obtained from swarming TrA plates and stained with tannic acid and silver nitrate.

Previous data indicated absence of swarm cells in the Δ*flhF* mutant of *B. cereus* grown on swarming-supporting media (Salvetti et al., 2007). In the same experimental conditions, we show that FlhF depletion also impairs cell migration. Indeed, as shown in **Figure 2A**, the Δ*flhF* mutant gave rise to colonies (4.2 ± 0.32 mm of diameter) that were significantly smaller than those produced by the swarming proficient wt (18.4 ± 1.28 mm of diameter; *P* = 0.0039) or Δ*flhF*-comp strain (14.2 ± 1.8 mm of diameter; *P* = 0.015). As expected, long and hyperflagellated swarmer cells were visualized in the colonies produced by the wt and the Δ*flhF*-comp strain only (**Figure 2B**).

### Effect of FlhF Depletion on Extracellular Proteins

To better characterize the effect of FlhF depletion on protein secretion by *B. cereus*, we performed comparative proteome analyses of the Δ*flhF* mutant, the wt, and the Δ*flhF*-comp strain grown in BHIG. There were no obvious growth differences between these strains in this medium, as already described (Salvetti et al., 2007). Additionally, as previously described (Salvetti et al., 2007), we again evaluated the activity of the cytosolic marker fructose-1,6-bisphosphate aldolase, a cytoplasmic enzyme that is not released by intact cells, in culture supernatants. No enzyme activity was detected. The bacterial supernatants were precipitated with TCA and analyzed using 2-DE. On average, 539 ± 103 spots were detected in the supernatant of the parental strain and 531 ± 99 spots in that derived from the Δ*flhF*-comp strain. This was not significantly different from the 430 ± 119 spots found in the mutant strain on average (*P* > 0.05). In the 2-DE gel analysis, 19 protein spots showed a different abundance of >1.5-fold (≥1.5 for upregulated proteins and ≤0.667 for downregulated proteins) in the Δ*flhF* mutant, compared to the wt (**Table 1**). These spots were identified by comparison with available gels (Gohar et al., 2002, 2005) and LC-MS/MS analysis. Three proteins were represented as multiple spots in the gel, having either similar mass but different isoelectric points or slight differences in the molecular mass due to putative post-translational modifications. No appreciable difference emerged between the wt and the Δ*flhF*-comp extracellular proteomes.

**Table 1:** *Bacillus cereus* ATCC 14579 supernatant proteins appreciably increased, reduced or absent in the supernatant of the Δ*flhF* (MP06) mutant following growth in BHIG broth.

Accession number	Functional annotation and designation^a^	Function category	MS score^b^	No of peptides^c^	Putative secretion or retention signals^d^	Ratio*^e^* MP06/wt	Ratio*^e^* MP07/wt
BC5101	Hemolysin, cereolysin O, **Clo**	Bacterial toxin	82	13	S	2.56	1.00
BC5445	Superoxide-dismutase, manganese-dependent **SodA**	Enzyme	77	4	NCS	2.44	1.00
BC5135	Enolase, **Eno**	Metabolism	80	10	TM	2.22	0.95
BC3874	Unknown (lipoprotein), **Unk_3**^∗^	Unknown	75; 73	15; 12	S	2.05; 1.39	1.00; 1.09
BC0295	60 kDa chaperone, **GroEL**	Folding, sorting and degradation	89	14	TM	1.85	1.00
BC1810	Enterotoxin Nhe, component B, **NHE_B_**	Bacterial toxin	84	9	S	1.82	1.00
BC0670	Phosphatidylcholine- specific phospholipase C, **PC-PLC**^∗^	Bacterial toxin, Enzyme	81; 78	8; 7	S	0.76; 0.36	0.95; 1.00
BC2735	Neutral protease, bacillolysin, **NPrP2**^∗^	Bacterial toxin, Enzyme	76; 82	16; 14	S	0.51; 0.21	1.10; 0.98
BC3761	Phosphatidylinositol-specific phospholipase C, **PI-PLC**	Bacterial toxin, Enzyme	76	9	S	0.47	0.91
BC1658	Flagellin, **Fla**	Cell motility	86	7	NCS	0.40	0.83
BC0671	Sphingomyelinase, **Smase**	Bacterial toxin, Enzyme	75	8	S	0.39	1.10
BC3104	Enterotoxin HBL, lytic component L_2_, **HBL-L_2_**	Bacterial toxin	83	14	S^†^	0.38	1.00
BC1179	Oligopeptide binding protein, **OppA_1**	Transporters	80	20	S, TM	0.32	1.25
BC1110	Hemolysin, cytotoxin K, **CytK**	Bacterial toxin	77	7	S	0.23	0.92
BC3616	Aconitate hydratase	Metabolism	79	21	TM	0.22	1.00
BC1657	Flagellin, **Fla**	Cell motility	86	7	NCS	0.22	1.11
BC0129	Elongation factor-Tu, **EF-Tu**	Translation factor	78	9	–	0	1.00
BC3161	Collagenase, **ColA**	Enzyme	80	12	S	0	1.00


*^a^The designations (short names) used for the various proteins are included. ^b^MS score is the score obtained by MASCOT. Protein scores greater than 70 are significant (*P* < 0.05). ^c^The number of peptides used to identify a protein. ^d^S: export signal peptide (SignalP 4.1 Server); TM transmembrane domain (TM pred); NCS: non-classically secreted protein (SecretomeP 2.0 Server). ^e^Ratio between the intensity of protein spots. ^∗^Proteins represented as multiple spots in the gels. ^†^No signal peptide was evidenced by the 4.1 version of SignalP Server. However, this protein has been reported as Sec-dependent (Fagerlund et al., 2010).*

In the Δ*flhF* mutant, the amount of six proteins increased and 12 proteins decreased in abundance compared to the parental strain (**Table 1**). The extracellular protein products significantly more abundant in the Δ*flhF* mutant included three predicted secretory proteins, cereolysin O (Clo; *P* = 0.007), the B component of NHE (NHE_B_; *P* = 0.041), both contributing to *B. cereus* pathogenicity (Lund and Granum, 1997; Alouf, 2000), and a protein with unknown function (*P* = 0.017). Three other proteins that do not exhibit a conventional signal peptide, the SodA superoxide-dismutase (*P* = 0.048), enolase (Eno; *P* = 0.007), and the chaperone GroEL (*P* = 0.01), were also present at higher levels in the supernatant of the Δ*flhF* mutant. Despite these proteins are cytosolic in nature, their orthologs are commonly detected in an extracellular form in many bacteria, *B. cereus* and other *Bacillus* species included (Nouwens et al., 2002; Braunstein et al., 2003; Lenz et al., 2003; Gohar et al., 2005).

Six predicted secretory proteins playing a role in *B. cereus* pathogenicity, since they are able to degrade phospholipids (PC-PLC; *P* = 0.017. PI-PLC; *P* = 0.0034. Smase; *P* = 0.0072), proteins (bacillolysin, NprP2; *P* = 0.032 and *P* = 0.007. Collagenase, ColA; *P* = 0.0002), and to form pores in planar lipid bilayers (cytotoxin K; *P* = 0.034), were significantly less abundant in the extracellular proteome of the Δ*flhF* mutant. The membrane-anchored substrate-binding protein OppA_1 of the oligopeptide permease transporter (Slamti et al., 2015) was also significantly reduced (*P* = 0.01) in the mutant secretome. In agreement with our previous results (Salvetti et al., 2007), the flagellin proteins BC1657 (*P* = 0.0091) and BC1658 (*P* = 0.016), classified as non-conventionally secreted proteins by the SecretomeP 2.0 server, were found significantly less abundant in the Δ*flhF* strain. In addition, a significant reduction of the L_2_ component of HBL (HBL-L_2_; *P* = 0.023), apparently lacking a signal peptide by the SIGNALIP 4.1 but herein classified as secretory accordingly to Fagerlund et al. (2010), was demonstrated for the mutant strain. The mutant also displayed a reduction in the spots corresponding to aconitate hydratase (BC3616; *P* = 0.0048) and elongation factor-Tu (EF-Tu; *P* = 0.002), commonly found in the cytoplasm.

### Contribution of FlhF to *B. cereus* Pathogenicity

**FIGURE 3:**
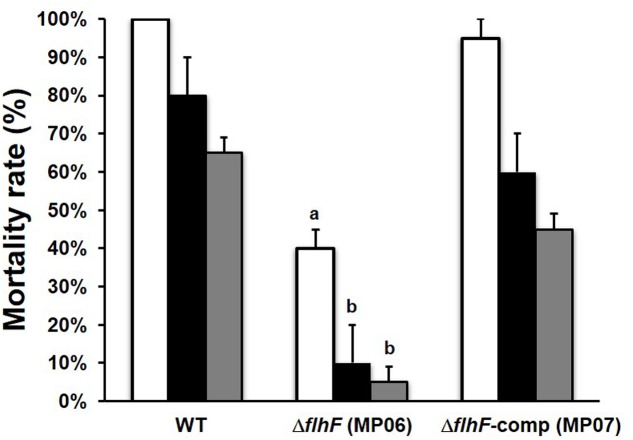
**Pathogenicity of *B. cereus* strains in *G. mellonella* larvae.** Mortality rates obtained after infection with 10^3^ (white bars), 10^2^ (black bars) and 10^1^ (gray bars) CFU per larva. ^a^*P* < 0.01; ^b^*P* < 0.05.

Based on the observation that several virulence proteins were less abundant in the supernatant of the Δ*flhF* mutant and that this strain was unable to swarm, we wondered whether the deficiency in FlhF would affect *B. cereus* pathogenicity in an *in vivo* model of infection. To this aim, larvae of the greater wax moth *G. mellonella*, which have been shown to provide useful insights into the pathogenesis of a wide range of microbial infections (Jander et al., 2000; Purves et al., 2010), were used to evaluate the pathogenicity of the wt, Δ*flhF*, and Δ*flhF*-comp strains. Larvae were intra-hemocoelically injected with three doses of mid-log phase bacteria and the number of dead larvae was recorded. As shown in **Figure 3**, a significant reduction in larvae mortality compared to the wt was observed when the Δ*flhF* mutant was inoculated at the three doses (*P* < 0.01 for the highest dose, and *P* < 0.05 for the other doses). No significant difference was observed in the mortality rates after injection of the wt or the Δ*flhF*-comp strain at different doses. As resulted from Probit analysis, the LD_50_ of the Δ*flhF* mutant was higher (3.16 ± 0.83 × 10^3^ CFU per larva) than the value obtained for the wt (9.10 ± 6.73 CFU per larva). The LD_50_ of the Δ*flhF*-comp strain was 6.27 ± 4.63 × 10^1^ CFU per larva.

To test whether differences in larvae mortality could be attributed to a defect in bacterial growth *in vivo*, the bacterial load in infected larvae was quantified over time. Larvae were infected with 10^4^ CFU of wt, Δ*flhF*, or Δ*flhF*-comp. At selected time points, the number of CFU per larva was determined. Infection of *G. mellonella* with all *B. cereus* strains resulted in an initial 10-fold reduction compared to the infectious dose in the CFU recovered from infected larvae at 2-h post infection (wt: 1755 ± 214.6 CFU/larva; Δ*flhF*: 1673 ± 350.4 CFU/larva; Δ*flhF*-comp: 1780 ± 280.2 CFU/larva). Bacterial recovery progressively increased over time up to 10000-fold the infectious dose at 24- h post infection (wt; 3.45 ± 0.102 × 10^8^ CFU/larva; Δ*flhF*: 3.48 ± 0.856 × 10^8^ CFU/larva; Δ*flhF*-comp: 3.46 ± 0.103 × 10^8^ CFU/larva). No significant difference in the CFU recovery of different strains was observed at each time point considered (data not shown). *B. cereus* was never recovered from non-infected larvae. Our data demonstrate that *B. cereus* wt, Δ*flhF*, and Δ*flhF*-comp are able to replicate at the same rate in *G. mellonella*.

## Discussion

Most of the information on the physiological role of FlhF comes from studies on polarly flagellated, monotrichous bacteria, in which the deletion of *flhF* results in the absence or mislocalization of flagella leading to swimming/swarming defects (Schuhmacher et al., 2015). In peritrichously flagellates, *flhF* orthologs are found in *Bacillus* and *Clostridium* species and data on FlhF function have been produced for *B. subtilis* and *B. cereus* (Carpenter et al., 1992; Zanen et al., 2004; Bange et al., 2007; Salvetti et al., 2007; Guttenplan et al., 2013). In both species, deletion of *flhF* leads to mislocalization of flagella and, in *B. cereus*, flagellar number as well as swimming motility are also reduced (Salvetti et al., 2007). FlhF has been suggested to target the first building block to the future flagellar assembly site by an unclear mechanism (Schuhmacher et al., 2015). The B-domain of FlhF seems to play an important role and differences within this domain might be important criteria to define different flagellation patterns in different species.

In this study, *in silico* analysis shows that, similar to Ffh and FtsY, *B. cereus* FlhF contains a conserved NG domain that includes the GTPase (G-domain) and the regulatory domain (N-domain). Besides the NG domain, FlhF contains an N-terminal domain of basic character (B-domain) that seems to be natively unfolded (**Figure 1A**), as already demonstrated for *B. subtilis* FlhF (Bange et al., 2007), but displays no homology with the same domain of other bacteria. This result is in line with the significant variations in size and conservation found in the B-domain among species, which suggested that this domain might exert species-specific functions (Schuhmacher et al., 2015).

SRP-GTPases are known to form dimers depending on the GTP load (Leipe et al., 2002; Gasper et al., 2006). While Ffh and FtsY aggregate to form a heterodimeric complex, FlhF act as a stable homodimer that bound GTP/GDP-Mg^2+^ in many organisms (Bange et al., 2007; Green et al., 2009; Schniederberend et al., 2013). SRPs dimerization capacity requires specific amino acid motifs that are typical of eukaryotic and bacterial SRP-GTPases (Anand et al., 2006) and constitute the active site. The presence of typical sequence motifs in *B. cereus* FlhF (**Figures 1B,C**) suggests that the protein forms a stable homodimer bound to GTP also in this organism.

By analyzing migration on TrA, an ideal medium for evaluating swarming migration by *B. cereus* ATCC 14579 (wt; Salvetti et al., 2011), we show that swarming motility is completely abolished in the Δ*flhF* mutant of *B. cereus*. This finding together with the previous demonstration that the mutant swimming motility was only reduced (Salvetti et al., 2007) suggests that other factors than flagella may be affected by the FlhF depletion. In fact, while swimming motility of cells primarily depends on flagella, additional factors are required for swarming. Perturbations in the flagellar motor, outer polysaccharides, osmotic agents, surfactants, as well as quorum-sensing signals have been shown to alter swarming in several bacteria (Kearns, 2010; Partridge and Harshey, 2013). Future studies will be required for defining the effects of FlhF deprivation on these factors.

Comparative proteomic analysis of culture supernatants indicated that the Δ*flhF* mutant of *B. cereus* displays an alteration in the quantity of certain extracellular proteins (**Table 1**). The amount of the major part of these proteins was lower in the proteome of the mutant compared to the wt and Δ*flhF*-comp strain, suggesting that FlhF can be involved in protein secretion, as already reported for *P. putida* (Pandza et al., 2000). In the SRP system, the SRP (Ffh)/4.5S RNA complex, in its GTP-bound form, attaches to the N-terminal sequence of the newly formed protein as it comes off the ribosome, and then docks with its receptor (FtsY) on the membrane, thus resulting in the insertion of the protein into the membrane (Akopian et al., 2013). *B. cereus* FlhF is homologous to both the SRP and the membrane receptor of this pathway and, as already suggested for other bacteria (Bange et al., 2007; Balaban et al., 2009), could play a role in either activities. FlhF has been shown to recruit the earliest flagellar component FliF and to insert itself in the cytoplasmic membrane in *V. cholerae* (Green et al., 2009). *B. cereus* FlhF could perform a similar function for flagellar proteins, but also for other proteins (PC-PLC, NPrP2, PI-PLC, Smase, HBL-L_2_, CytK) that need to be directed to Sec translocase to be exported outside the cell. Nevertheless, although less pronouncedly, these proteins are still present in the secretome of the Δ*flhF* mutant, suggesting that, if FlhF acts as SRP, protein targeting to the membrane can still occur even in the absence of functional FlhF and that other systems may act in the export of these proteins. Although the secretion defects observed in the absence of functional FlhF might be due to its role as SRP-like protein, an effect of FlhF deprivation on regulatory pathways shared between the genes encoding the altered proteins cannot be excluded.

*Galleria mellonella* was previously shown to be a good alternative to the mouse model for evaluating the virulence of *B. cereus* (Salamitou et al., 2000). The virulence potential of *B. cereus* is due to the secretion of a plethora of virulence proteins, but also to the presence of flagella, which play a crucial role in swimming and swarming motility and in bacterial adhesion to surfaces. Infection of *G. mellonella* with a *B. cereus* mutant in the Smase gene was previously shown to cause a reduction in larvae mortality, while mutations in *nheBC* had a little impact on *G. mellonella* survival (Doll et al., 2013). Infection with a *B. cereus* mutant in *codY*, encoding a positive regulator of the PC-PLC gene and many other virulence genes (Frenzel et al., 2012; Slamti et al., 2015), as well as with swimming defective mutant (Frenzel et al., 2015), drastically reduced larvae mortality. *G. mellonella* infection with a *B. thuringiensis* strain unable to swarm and to secrete of HBL-L_2_ resulted in a strong reduction of larvae mortality (Bouillaut et al., 2005). In this study, we demonstrate that lack of FlhF substantially reduces *B. cereus* pathogenicity in *G. mellonella*. Since no difference with the wt in the replication of the Δ*flhF* mutant in *G. mellonella* was found, we can assume that the reduced pathogenicity of the mutant is due to the different virulence potential this strain shows. Other than being less flagellated and unable to swarm, the mutant strain shows a reduction in the secretion of PC-PLC, Smase, HBL-L_2_, and many other virulence proteins (PI-PLC, CytK and ColA) (Beecher et al., 2000; Bottone, 2010; Ramarao and Sanchis, 2013). Despite the amount of some virulence proteins (NHE_B_ and Clo) is increased in the secretome of the Δ*flhF* mutant, we can speculate that these factors have a minor impact on *B. cereus* pathogenicity in *G. mellonella*. Therefore, the reduced production of some virulence proteins, of swimming, and the lack of swarming motility appear responsible of the scarce pathogenicity exerted by the Δ*flhF* mutant in *G. mellonella*.

## Conclusion

The flagellar protein FlhF, which controls the number and localization of flagella in *B. cereus* (Salvetti et al., 2007), is essential for swarming motility and full virulence. FlhF might act directly or through regulatory mechanisms on protein secretion or synthesis. Protein-protein interaction studies and partial gene deletion mutants will help in clarifying the molecular mechanisms underlying its peculiar activity.

## Author Contributions

All authors listed, have made substantial, direct and intellectual contribution to the work, and approved it for publication.

## Conflict of Interest Statement

The authors declare that the research was conducted in the absence of any commercial or financial relationships that could be construed as a potential conflict of interest.

## Footnotes

1 http://blast.ncbi.nlm.nih.gov/Blast.cgi

2 http://www.uniprot.org/

3 http://prodom.prabi.fr/prodom/current/html/home.php

4 http://www.sbg.bio.ic.ac.uk/phyre2

5 http://raptorx.uchicago.edu/StructurePrediction/predict/

6 http://www.matrixscience.com

7 http://www.genome.jp/kegg/

8 http://www.cbs.dtu.dk/services/SignalP/

9 http://www.cbs.dtu.dk/services/TatP/

10 http://www.cbs.dtu.dk/services/SecretomeP/

11 http://www.ch.embnet.org/software/TMPRED_form.html
